# Transcriptional profiles discriminate bone marrow-derived and synovium-derived mesenchymal stem cells

**DOI:** 10.1186/ar1827

**Published:** 2005-09-20

**Authors:** Farida Djouad, Claire Bony, Thomas Häupl, Gilles Uzé, Najiba Lahlou, Pascale Louis-Plence, Florence Apparailly, François Canovas, Thierry Rème, Jacques Sany, Christian Jorgensen, Danièle Noël

**Affiliations:** 1 INSERM Unit 475, Montpellier, France; 2 Rheumatology, Charité Hospital, Berlin, Germany; 3 CNRS, UMR 5124, Montpellier, France; 4 Hormonal Biology Laboratory, St Vincent de Paul Hospital, Paris, France; 5 Immuno-Rhumatologie, Lapeyronie Hospital, Montpellier, France

## Abstract

Previous studies have reported that mesenchymal stem cells (MSC) may be isolated from the synovial membrane by the same protocol as that used for synovial fibroblast cultivation, suggesting that MSC correspond to a subset of the adherent cell population, as MSC from the stromal compartment of the bone marrow (BM). The aims of the present study were, first, to better characterize the MSC derived from the synovial membrane and, second, to compare systematically, in parallel, the MSC-containing cell populations isolated from BM and those derived from the synovium, using quantitative assays. Fluorescent-activated cell sorting analysis revealed that both populations were negative for CD14, CD34 and CD45 expression and that both displayed equal levels of CD44, CD73, CD90 and CD105, a phenotype currently known to be characteristic of BM-MSC. Comparable with BM-MSC, such MSC-like cells isolated from the synovial membrane were shown for the first time to suppress the T-cell response in a mixed lymphocyte reaction, and to express the enzyme indoleamine 2,3-dioxygenase activity to the same extent as BM-MSC, which is a possible mediator of this suppressive activity. Using quantitative RT-PCR these data show that MSC-like cells from the synovium and BM may be induced to chondrogenic differentiation and, to a lesser extent, to osteogenic differentiation, but the osteogenic capacities of the synovium-derived MSC were significantly reduced based on the expression of the markers tested (collagen type II and aggrecan or alkaline phosphatase and osteocalcin, respectively). Transcription profiles, determined with the Atlas Human Cytokine/Receptor Array, revealed discrimination between the MSC-like cells from the synovial membrane and the BM-MSC by 46 of 268 genes. In particular, activin A was shown to be one major upregulated factor, highly secreted by BM-MSC. Whether this reflects a different cellular phenotype, a different amount of MSC in the synovium-derived population compared with BM-MSC adherent cell populations or the impact of a different microenvironment remains to be determined. In conclusion, although the BM-derived and synovium-derived MSC shared similar phenotypic and functional properties, both their differentiation capacities and transcriptional profiles permit one to discriminate the cell populations according to their tissue origin.

## Introduction

Mesenchymal stem cells (MSC) are progenitor cells that have the potential to differentiate into lineages of mesenchymal tissues including cartilage, bone, muscle and fat. They were initially isolated from bone marrow (BM) and characterized by the expression of various cell surface markers [1,2]. MSC have more recently been obtained from adipose tissue, peripheral blood, cord blood, cartilage [3-6] and synovial tissue [7].

Identification of MSC in the synovium has raised speculations about their biological role in the normal or pathologic joint physiology. As MSC have a great potential to repair damaged tissues, they are likely to contribute to joint regeneration in arthritis. Indeed, MSC have been detected in the synovial fluid of patients with arthritis, with a higher prevalence in osteoarthritis (OA). In this OA, MSC may participate in the highly active process of regeneration due to the reactivation of endochondrial ossification in the advanced phase of the disease [8]. However, a significant reduction in the *in vitro *chondrogenic and adipogenic activities of MSC has been reported in patients with OA [9]. The authors suggest that changes in the differentiation profile of MSC account for the increase of bone density and loss of cartilage that are characteristics of OA. Recent data suggest a possible involvement of MSC in the pathophysiology of OA, but also in inflammatory arthritis [10]. In the study, the authors show that during the induction phase of collagen-induced arthritis, marrow-derived mesenchymal cells accumulate in the synovium preceding the clinical onset of arthritis and afflux of inflammatory cells [10]. Thus, although still to be demonstrated, MSC may play a pivotal role in the induction phase of arthritis by promoting the accumulation of immunocompetent cells into the joint.

To date, identification of MSC from the synovial membrane exclusively relies on their phenotypic characterization and on the assessment of their differentiation potential. MSC from the synovial membrane were shown to express various surface markers (CD9, CD10, CD13, CD44, CD54, CD55, CD90, CD105, CD166, D7-FIB) and to be negative for CD14, CD20, CD45 and CD133 by fluorescent-activated cell sorting (FACS) analysis [7,8,11]. A more detailed study involving molecular characterization of MSC from the synovial membrane by RT-PCR has revealed the expression of various matrix molecules, adhesion molecules, ligands, receptors and transcription factors [7]. Functional characterization of MSC from the synovial membrane has shown their multilineage potential as they are able to differentiate towards chondrocytes, osteoblasts, adipocytes and, to a lesser extent, towards myocytes [7].

Isolation of MSC from the synovium [7,11], mainly based on adhesion properties, relies on the technique used to isolate synovial fibroblasts, suggesting that only a subset of the cell population corresponds to the MSC. On the basis of the present knowledge of the biology of BM-derived MSC, we underwent parallel studies to phenotypically and functionally compare the MSC isolated from both tissues. Interestingly, using quantitative analyses, our results show that the potential of differentiation towards osteocytes were significantly reduced in synovium-derived MSC. The present study is the first to demonstrate that MSC from the synovial membrane share the same immunosuppressive features as BM-MSC because they are able to inhibit the T-cell proliferation in a mixed lymphocyte reaction (MLR) and to display indoleamine 2,3-dioxygenase (IDO) activity. Importantly, using macroarray technology we provide evidence that the transcriptional profiles could be used to discriminate the MSC by function of their tissue origin, Activin A being one major upregulated gene in BM-MSC.

## Materials and methods

### Cell culture

Human MSC cultures were established from BM aspirates of healthy donors or from OA patients and rheumatoid arthritis (RA) patients undergoing hip replacement surgery, after informed consent. The cell suspension was diluted in serum-free medium, filtered on a nylon membrane (Cell Strainer; Dutscher, Cergy, France) and centrifuged at 200 × *g *for 10 min at ambient temperature. Mononuclear cells were then plated at the density of 5 × 10^4 ^cells/cm^2 ^in α-MEM, supplemented with 10% fetal bovine serum (Perbio Science France SAS, Brebières, France), 1 ng/ml basic fibroblast growth factor, 100 U/ml penicillin and 100 μg/ml streptomycin. When cultures reached near confluence, cells were detached with 0.05% trypsin and 0.53 mM ethylenediamine tetracetic acid, and were subsequently replated at the density of 1,000 cells/cm^2^. BM adherent cells were used between passage 2, when a homogeneous population of cells was microscopically observed, and passage 7. The median age of the BM-MSC samples was 53.13 ± 18.3 years, corresponding one-half to healthy donors and approximately one-quarter to RA patients and one-quarter to OA patients.

Human synovium-derived adherent cells were isolated from synovial tissues either post mortem (healthy donors) or at the time of surgical knee replacement for degenerative OA or RA. Synovial tissues were finely minced and digested with 0.2% collagenase in DMEM containing 10% fetal bovine serum, 100 U/ml penicillin and 100 μg/ml streptomycin (complete DMEM). Following overnight incubation at 37°C, cells were collected by centrifugation, washed once and filtered on a nylon membrane (Cell Strainer; Dutscher). The cell suspension was then plated in complete DMEM in a 75 cm^2 ^flask and passaged when reaching near confluence according to previous report [7]. Synovial cells were used between passage 4 and passage 8. The median age of synovium-derived MSC samples was 54.6 ± 28.8 years, corresponding one-half to RA patients and approximately one-quarter to OA patients and one-quarter to healthy donors. Due to ethical considerations, BM-derived and synovium-derived cells were not obtained from the same patients.

### Phenotypic characterization

For flow cytometry, cells were harvested by treatment with 0.05% trypsin and 0.53 mM ethylenediamine tetracetic acid, and were resuspended in PBS containing 0.1% BSA and 0.01% sodium azide. Cell aliquots (10^5 ^to 5 × 10^5 ^cells/100 μl) were incubated on ice with conjugated mAbs against CD14, CD34, CD44, CD45, CD73, CD90 and CD105 (BD Pharmingen, Le Pont de Claix, France) or conjugated isotypic controls. Flow cytometry was performed on a fluorescence-activated cell sorter (FACS Scan, BD Biosciences, Le Pont de Claix, France), and data were analyzed with the Cellquest software (BD Pharmingen).

For immunofluorescence analysis, cells were fixed with acetone:methanol (1:1), washed with PBS and incubated with the primary mAb specific for the human prolyl-4-hydroxylase (Dako, Trappes, France) at 1:50 dilution for 30 min at room temperature. Washed slides were then incubated with a secondary fluorescein isothiocyanate-conjugated goat anti-mouse antibody for 30 min in the dark. Fluorescence was visualized using a Zeiss standard microscope equipped with an AxioCam MRcamera (Carl Zeiss Vision, Le Pecq, France).

### Induction of genes by interferons

Cells were cultured in the presence of 1,000 U/ml IFN-α, IFN-β or IFN-γ at 37°C for 6 or 48 hours in the case of IFN-γ. Expression of major histocompatibility complex (MHC) class I and class II molecules was detected by flow cytometry using mAbs specific for HLA-A, HLA-B, HLA-C and anti-HLA-DR molecules (W6.32 and L243 clones, respectively). Induction of the 6–16 gene rapidly induced by IFNs was recorded by quantitative real-time PCR as previously described [12].

### Chondrogenic differentiation and osteogenic differentiation

Chondrogenic differentiation was induced by a 21-day culture in micropellet. Briefly, cells (2.5 × 10^5 ^cells) were pelleted by centrifugation in 15 ml conic tubes and cultured in BMP-2-conditioned chondrogenic medium. Osteogenesis was induced by culture at low density (1.5 × 10^4 ^cells in a 100-mm-diameter culture dish) for 21 days in BMP-2-conditioned osteogenic medium. The conditioned media were obtained after incubation of C9 cells at confluence for 48 hours in the presence of either chondrogenic medium or osteogenic medium. C9 cells are derived from the C3H10T1/2 murine MSC line, and they express 1,230 ng hBMP-2 per 24 hour/10^6 ^cells under the control of a TetOff promoter [13]. As the control, supernatants from C3H10T1/2 cells were unable to induce any cell differentiation (data not shown). The chondrogenic medium consisted of DMEM supplemented with 0.1 μM dexamethasone (Sigma, l'Isle d'Abeau, France), 0.17 mM ascorbic acid and 1% insulin-transferrin-sodium selenite media supplement (Sigma). The osteogenic medium consisted of DMEM medium supplemented with 10% FCS, 10 mM β-glycerophosphate (Sigma), 0.1 μM dexamethasone (Sigma) and 0.05 mM ascorbic acid (Sigma). The adipogenic differentiation potential for BM-derived and synovium-derived MSC has been checked according to a previously described protocol [14].

### Real-time RT-PCR

Total RNA was extracted from cell micropellets using the RNeasy mini kit (Qiagen S.A., Courtaboeuf, France) and from cells in monolayers using the Promega SV Total RNA Isolation System protocol (Promega, Charbonnières-les-bains, France) as recommended by the suppliers. Total RNA was reverse transcribed using Multiscribe reverse transcriptase (Applied Biosystems, Courtaboeuf, France). The TaqMan gene expression arrays and the TaqMan Universal Master Mix were used according to the manufacturer's recommendations (Applied Biosystems). Measurement and analysis of gene expression were performed using the ABI Prism 7000 Sequence Detection System software (Applied Biosystems). Content of cDNA samples was normalized by subtracting the number of copies of the endogenous GAPDH reference gene from the number of copies of the target gene (ΔCt = Ct of target gene – Ct of GAPDH). Expression of the specific gene was calculated using the formula 2^-(ΔCt)^.

### Mixed lymphocyte reaction

MLRs were performed as previously described [15]. Briefly, splenocytes from BALB/c mice and DBA/1 mice were isolated and stimulator splenocytes were inhibited to proliferate by treatment with 50 μg/ml mitomycin C (Sigma) at 37°C for 45 min. Each responder cell population and each stimulator cell population was seeded in triplicate at the concentration of 10^5 ^cells/100 μl per well, in 96-well round-bottom plates (BD Biosciences, Le Pont de Claix, France). Synovium-derived and BM-derived adherent cells (10^5 ^cells) were added to the MLR to obtain a 300 μl final volume. After 3 days of incubation, 1 μCi/well [^3^H]thymidine was added overnight and thymidine incorporation was measured using a β-scintillation counter. Each experiment was performed at least three times.

### IDO activity measurement

Cells were stimulated with IFN-γ (1,000 U/ml) and/or tumor necrosis factor alpha (TNF-α) (50 ng/ml) for 48 hours in DMEM supplemented with L-tryptophan (100 μg/ml). IDO enzyme activity was measured by tryptophan-to-kynurenine conversion with photometric determination of the kynurenine concentration in the supernatant as the readout, as previously reported [16]. Briefly, 160 μl cell supernatant were transferred to a 96-well culture plate and 10 μl of 30% trichloroacetic acid was added for 30 min at 50°C. After centrifugation, 100 μl supernatant was mixed with 100 μl freshly prepared Ehrlich's solution and the absorbance was read with a microplate reader at 450 nm.

### Isolation of total RNA and cDNA hybridization

Total RNAs of adherent cells (four separate samples from healthy BM and four separate samples from healthy synovium between passage 4 and passage 6) were extracted using the RNeasy mini kit (Qiagen S.A.) according to the manufacturer's instructions. Radiolabeled cDNA was prepared from each RNA sample with the Atlas array kit (Clontech, Saint Quentin en Yvelines, France) by a reverse-transcription step in the presence of α-[^32^P]dATP. The radiolabeled samples were hybridized to the Human Cytokine/Receptor Atlas Nylon cDNA Expression Array (BD Biosciences). After stringent washes, membranes were scanned using a Phosphoimager (Amersham Pharmacia Biotech, Saclay, France).

### Gene array analysis

Quantification was performed using the AtlasImage software (BD Biosciences). Data from each array were normalized by the median value to eliminate the variability due to the sample labeling or the exposure duration. The normalized median was arbitrarily given the value 150. Analysis was performed using the Cluster and TreeView hierarchical clustering software developed by Eisen and colleagues [17]. Two filters have been used: one filter aimed at retaining only genes expressed above the median value, and the second filter retained genes for which the difference between the maximum and minimum values was twice the median value. Data were log-transformed (log-base 2), and the genes were median centered and clustered by correlation average linkage clustering. The hierarchical clustering was visualized with TreeView.

### Activin A quantification by ELISA

Total activin A was measured by means of a highly specific solid-phase enzyme-linked immunometric assay using reagents supplied by DSL-France (Cergy-Pontoise, France). The first antibody was an anti-βA-subunit monoclonal antibody immobilized on microplate wells. The second antibody was a biotinylated monoclonal antibody. In order to minimize matrix effects, the assay procedure was adapted to the culture medium: the assay standards were reconstituted with nonincubated medium, which was also used as a diluent. The assay had no detectable cross-reaction with inhibin A, follistatin, activin B or inhibin B. The dilution curves of high-level samples paralleled the standard curves. The sensitivity was <0.1 ng/ml and the inter-assay coefficient of variation was <10%.

### Statistical analysis

Statistics were performed with the Student *t *test or an unpaired Mann-Whitney test as appropriate according to data distribution. All data were analyzed by the program Instat (Graphpad, San Diego, CA, USA).

## Results

### Phenotypic characterization

**Figure 1:**
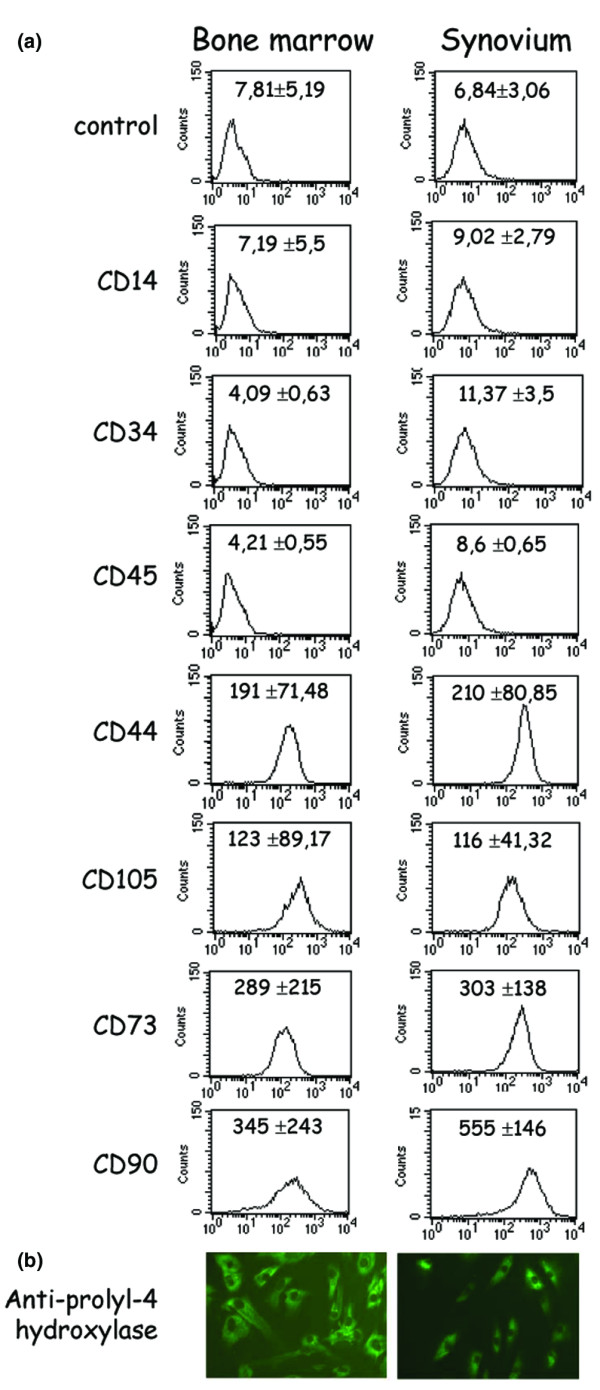
Immunophenotype of bone marrow-derived (left) and synovium-derived adherent cells (right). **(a) **Fluorescence-activated cell sorting of CD marker expression. Results are expressed as the mean fluorescence intensity ± standard error of the mean. One representative experiment out of six different samples of bone marrow mesenchymal stem cells (three normal, two osteoarthritis and one rheumatoid arthritis) and synovium mesenchymal stem cells (two rheumatoid arthritis, two osteoarthritis and two healthy) is shown. Control corresponds to the fluorescence due to the isotypic control. **(b) **Immunofluorescence staining on cells in a monolayer using the monoclonal antibody specific for human prolyl-4-hydroxylase.

Adherent cells isolated from BM or synovial tissue were first characterized according to the expression of surface markers known to be expressed or absent on BM-MSC. By FACS analysis, we showed that more than 99% of these cells from both tissues were negative for the expression of CD14, CD34 and CD45 and were positive for CD44, CD73, CD90 and CD105 (Fig. 1a). Similar results were also obtained using human primary skin fibroblasts (data not shown). The fluorescence intensities for each marker were not statistically different between the two cell populations, suggesting a similar level of expression on both cell populations – except for the marker CD90 (*P *= 0.0311), which was higher on MSC from synovium. Because contradictory results were reported using the antibody specific for prolyl-4-hydroxylase to immunophenotype the MSC [18,19], we checked whether it could be useful to discriminate between MSC derived from BM or MSC derived from synovium. Although no expression was found on both cell populations by FACS analysis (data not shown), they were both positive in immunocytochemistry (Fig. 1b). Similarly, the skin fibroblast cells used as the control were also positive for this marker (data not shown). Indeed, using various markers, adherent cell populations isolated from the two tissues displayed a similar phenotype, commonly observed with MSC-containing cell populations derived from BM.

### Expression of MHC class I and class II

**Figure 2:**
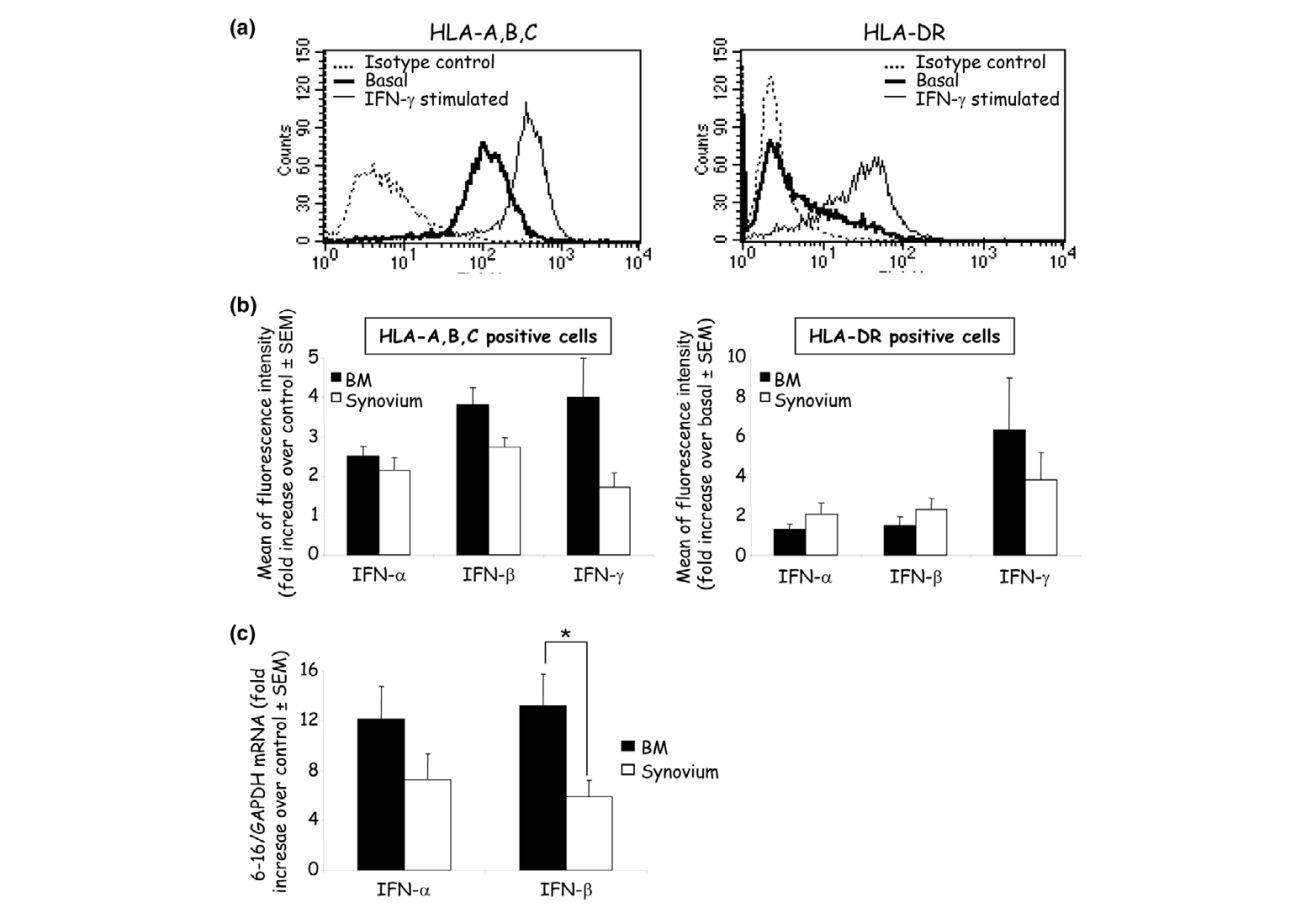
Induction of HLA class I and class II expression by IFN in adherent cells. **(a) **One example of basal and IFN-γ-induced expression of HLA-A, HLA-B, HLA-C (left) and HLA-DR (right) molecules on mesenchymal stem cells from bone marrow (BM) by fluorescence-activated cell sorting analysis. **(b) **Expression of HLA-A, HLA-B, HLA-C (left) and HLA-DR (right) molecules by BM-derived cells (*n *= 5; two normal, two osteoarthritis and one rheumatoid arthritis) and synovium-derived cells (*n *= 7; three rheumatoid arthritis, two normal, two osteoarthritis) after induction with 1,000 U/ml IFN-α, IFN-β and IFN-γ, shown as the fold increase of the mean fluorescence intensity over the control ± standard error of the mean. **(c) **Detection of the 6–16 early response gene to IFN, assessed by quantitative RT-PCR, normalized to GAPDH mRNA (*n *= 3 for synovium-derived and *n *= 4 for BM-derived cell samples). Results display the fold increase of the 6–16 gene in IFN-induced samples over nontreated samples. * *P *< 0.05.

BM-MSC are known to be positive for MHC class I molecules and to be negative for MHC class II molecules in basal culture conditions, both being upregulated following treatment with IFN-γ [1]. We confirmed these observations with the cells isolated from BM used in this study (Fig. 2a) and with the synovium-derived cells (data not shown) as already reported [20]. However, no data are available on the effect of IFN-α and IFN-β on the expression of MHC molecules on synoviocytes or BM-MSC. We showed that, similarly to IFN-γ, IFN-α and IFN-β significantly upregulated the MHC class I molecules in both cell populations. Only IFN-γ significantly induced the expression of class II molecules, and both the number of positive cells (56 ± 16% and 36 ± 17% for BM-derived and synovium-derived cells, respectively) and their mean fluorescence were increased (Fig. 2b). However, no statistically significant difference between cells isolated from the two tissues was observed.

We then investigated whether the 6–16 gene, which is a representative of the early type I IFN-stimulated genes, was also upregulated in adherent cells isolated from BM or synovium. The expression of the 6–16 gene was upregulated with IFN-α and IFN-β (Fig. 2c), and to a lesser extent with IFN-γ (data not shown), and a statistically reduced expression level was observed with cells isolated from the synovial membrane. Thus, for most of the markers examined, no significant difference between adherent cells from BM and synovium was observed in our culture conditions.

### Differentiation capacities

**Figure 3:**
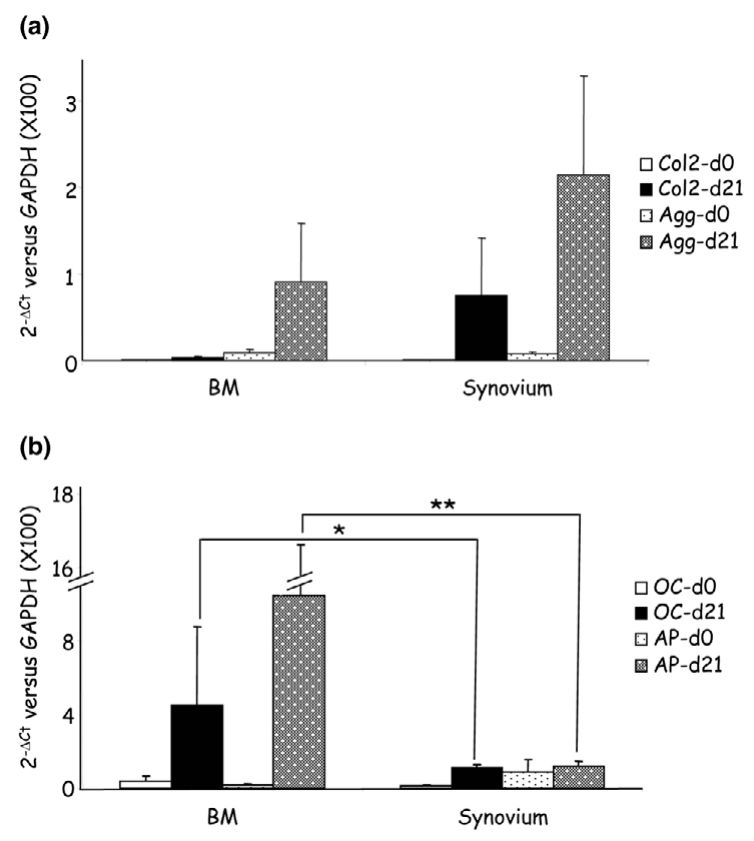
*In-vitro*-induced differentiation of bone marrow-derived and synovium-derived adherent cells. **(a) **Chondrogenic differentiation was evaluated after 21 days in micropellet culture. Expression of the specific markers for chondrogenesis (collagen type II [Col2] and aggrecan [Agg]) was determined by quantitative RT-PCR from the various samples: eight bone marrow (BM) mesenchymal stem cells (MSC) (four healthy, two osteoarthritis [OA], two rheumatoid arthritis [RA]) and eight synovium-MSC (four RA, two OA, two RA). **(b) **Osteogenic differentiation was evaluated after 21 days in monolayers. Expression of the osteogenic markers (osteocalcin [OC] and alkaline phosphatase [AP]) was determined by quantitative RT-PCR from the various samples: eight BM-MSC (four healthy, two OA, two RA) and eight synovium-MSC (four RA, two OA, two healthy). The expression levels were normalized on the basis of GAPDH expression, and the results are reported as ratios of the marker gene versus GAPDH using the formulae 2^-ΔCt ^(×100) ± standard error of the mean. Statistics compared eight cell samples from BM and eight cell samples from the synovium. * *P *< 0.05, ** *P *< 0.01.

Similarly to BM-MSC, synovial-derived MSC have been reported to differentiate into chondrocytes, osteocytes and adipocytes [7]. We compared the expression levels of specific markers following the induction of chondrogenesis or osteogenesis *in vitro *to accurately quantify the differentiation capacities of adherent cells obtained from the two tissues. The expression of two chondrogenic markers (collagen type II and aggrecan) and two osteogenic markers, (osteocalcin and alkaline phosphatase) was quantified relative to GAPDH by quantitative RT-PCR (Fig. 3a, b). A significant increase of the mRNA was observed on both cell populations and for all the differentiation markers tested. The mean increase of collagen type II and aggrecan was stronger in synovium-derived cells than in BM-derived cells but the differences were not significant. Notably, the collagen type II marker was induced in all cell samples isolated from BM whereas only five out of eight samples from the synovial tissue were positive after differentiation (data not shown). Inversely, the mean induction of osteocalcin and alkaline phosphatase was statistically higher in cells from BM than in cells from the synovium. In these conditions, the primary skin fibroblastic cells were unable to differentiate along the chondrogenic and osteogenic pathways (data not shown). Although a high heterogeneity in the induction of the various markers was observed between the samples, synovial cells displayed a reduced osteogenic capacity.

### Immunosuppressive nature

**Figure 4:**
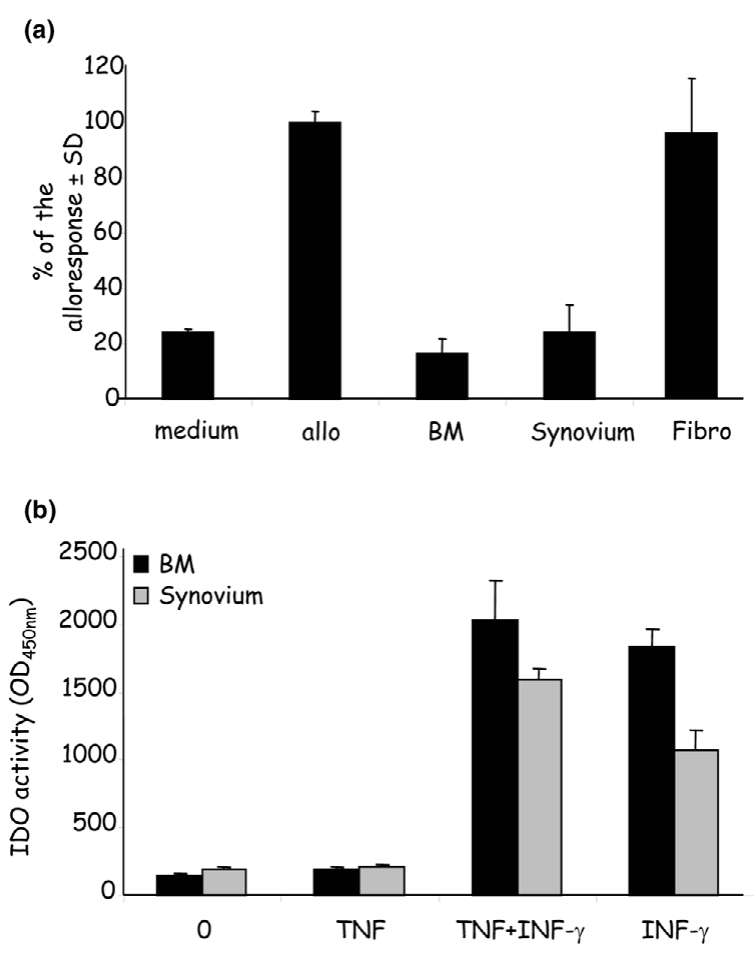
Immunosuppressive properties of bone marrow-derived and synovium-derived adherent cells. **(a) **Proliferative activity of T cells in a mixed lymphocyte reaction. Responding BALB/c splenocytes (10^5 ^cells) were incubated for 4 days with mitomycin-treated DBA/1 splenocytes (10^5^) in the presence or absence of 10^5 ^adherent cells from bone marrow (BM) (*n *= 10; six healthy, two rheumatoid arthritis, two osteoarthritis) or synovium (*n *= 10; five rheumatoid arthritis, two osteoarthritis, three normal) or normal fibroblasts (fibro; *n *= 3). The proliferative response corresponding to the average counts per minute of triplicates of alloreactive T cells (allo) was assigned the value of 100% ± standard deviation (SD). **(b) **The indoleamine 2,3-dioxygenase (IDO) activity was detected as the tryptophan to kinurenin conversion measured at an optical density (OD) of 450 nm. Cells were cultured in the absence or presence of IFN-γ and/or tumor necrosis factor alpha (TNF-α) for 48 hours and the detection of kinurenin was measured by photometry. The IDO activity is expressed as the mean activity from four different samples of cells from each tissue ± SD.

We and others have previously shown that BM-MSC exhibit immunosuppressive properties able to inhibit the proliferation of T cells in a MLR [15,21]. However, nothing was known on the behavior of synoviocytes. Similarly to BM-MSC, we showed that MSC from the synovium were able to suppress the proliferative capacities of T cells in a MLR whereas primary skin fibroblasts display no suppressive properties (Fig. 4a). It has very recently been reported that this effect may be mediated by the induction of the IDO activity in BM-MSC [22]. We thus determined whether cells derived from the synovium or from BM may display a similar induction of IDO activity upon IFN activation. As shown in Fig. 4b, incubation of cells with IFN-γ but not with TNF-α resulted in a similar induction of IDO activity in the two cell populations.

### Identification of genes differentially expressed

**Figure 5:**
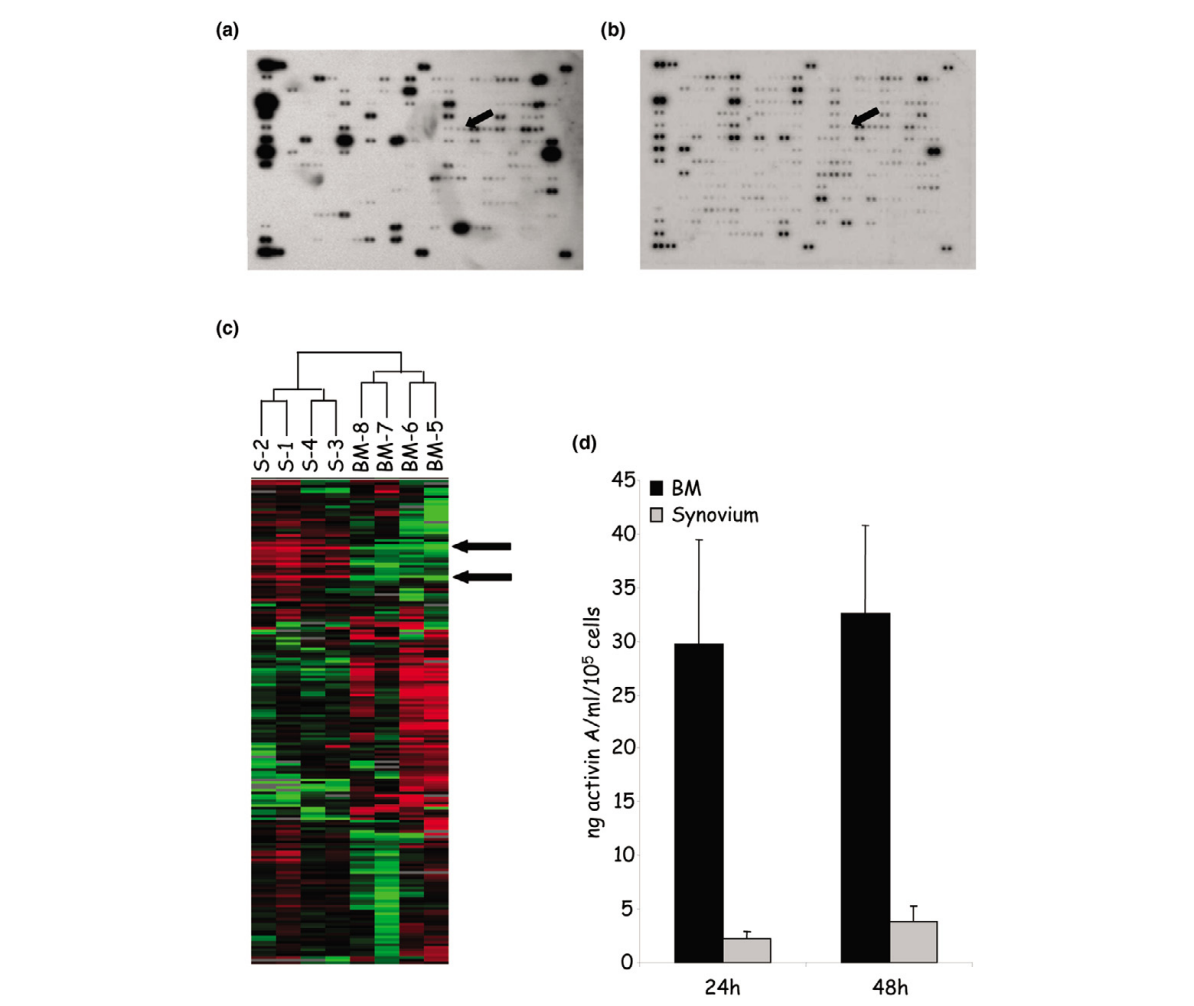
Analysis of genes differentially expressed by normal bone marrow-derived and synovium-derived adherent cells. Total RNAs were extracted from cells at passages 4–6 obtained from healthy donors and reverse-transcribed to radiolabeled cDNAs before hybridization to gene array membranes (Atlas Human Cytokine/Receptor Array; BD Biosciences). Each gene is represented as duplicate spots. **(a) **One example of a representative array from cells isolated from bone marrow (BM) (*n *= 4). The arrow represents the activin/inhibin βA subunit. **(b) **One example of a representative array from synovial cells (*n *= 4). The arrow represents the activin/inhibin βA subunit. **(c) **Hierarchical clustering analysis. The dendrogram at the top represents the relationship of the samples according to the similarity in their gene expression profile. Arrows indicate some areas of genes that permit one to discriminate between samples. Red, high expression, green, low expression. **(d) **Quantification of the secreted activin A by specific ELISA. Results are expressed as the mean of secretion in 24-hour and 48-hour supernatants of four cell samples ± standard error of the mean.

We then compared the cDNA expression profiles of these cell populations using the Atlas Human Cytokine/Receptor Array membrane. Radiolabeled cDNAs of four BM-derived cells versus four synovium-derived cells from healthy donors were hybridized on the membranes, which are composed of cDNAs from 268 genes (Fig. 5a, b). Data were analyzed using the Cluster and TreeView hierarchical clustering software, resulting in a color-coded gene expression representation: expression below the median is green, expression above the median is red, whereas the median expression across all samples is black. The software groups genes by similarities in their pattern of expression, and groups samples by similarities in their pattern of expression over all samples (Fig. 5c).

The analysis of the 268 genes thus ended with the classification of samples into two groups differentiating between the two tissue origins, BM or synovial tissue, suggesting that the differential expression of some genes should permit one to discriminate between the two cell populations (Table 1). The different gene expression patterns allowed us to focus on genes grouped in clusters whose expression was higher in either group. Among the genes that were overexpressed in cells isolated from BM, we checked for the expression of the βA subunit of inhibin or activin. The βA chains either homodimerize by disulfide bonding to form activin A or heterodimerize with inhibin α-chains to form inhibin A [23]. We thus used a specific ELISA developed to quantify the protein levels expressed by adherent cells isolated from the two tissues. Indeed, we specifically detected high levels of activin A in the supernatants from BM-derived cells and detected lower amounts from cells cultured from the synovial membrane (<1/10) (Fig. 5d).

## Discussion

It is now established that MSC can be isolated from the synovial membrane [7] as well as from BM or other tissues (for a review, see [1]). The culture conditions, based in part on adherence to plastic, used to isolate MSC from the synovial membrane are similar to those used to obtain fibroblastic synoviocytes, suggesting that only a subset of cells in this synovium-derived cell population are stem cells, like MSC from BM-derived cultures. As no specific marker for MSC is presently available, their characterization relies essentially on their functional properties, but to date no quantitative data allow comparison between MSC isolated from various tissues. In the present study we have shown that BM-derived and synovium-derived adherent cell populations can be induced to differentiate towards chondrocytes and osteoblasts, but a significant fivefold to 10-fold reduction in the expression levels of the osteogenic markers was observed with the adherent cells from the synovium. Furthermore, we show that the transcriptional profiles permit one to discriminate between the cell populations isolated from BM or the synovium, and that activin A might be a useful marker since it is highly secreted by MSC from BM.

In agreement with other studies [8,11], we confirm that, together with skin fibroblasts, synovium-derived adherent cells express various markers known to be present on BM-MSC, such as CD44, CD90 and CD105. We now show that the cells isolated from the synovial membrane also express the CD73 marker, an ecto-5'-nucleotidase recognized by the SH3 antibody, and express prolyl-4-hydroxylase. Prolyl-4-hydroxylase was shown to be expressed by synoviocytes [18,24] but to be absent on BM-MSC using FACS analysis [18]. In the present study we were able to detect the protein in both cell populations by immunocytochemistry, whereas it was negative by FACS analysis, suggesting that this marker was not specific for synoviocytes. Expression of MHC class I molecules on BM-MSC and synovial fibroblasts has been reported, as well as the induction of MHC class II molecules upon treatment with IFN-γ [25,26]. However, although IFN-β has been shown to induce the expression of MHC class I and class II molecules on various cell types [27,28], no data were available on the potential role of IFN-α and IFN-β on synovial fibroblasts or BM-MSC. To our knowledge, this is the first report to show that IFN-α and IFN-β upregulate the MHC class I molecules and the 6–16 gene, which is one of the early responsive genes induced by IFN, but fail to induce the MHC class II molecules on both cell populations. Altogether, these data illustrate the high similarity between adherent cell populations isolated from BM and the synovium, based on the expression of various phenotypic markers known to be currently tested with MSC. This suggests that these markers correspond to molecules expressed on cells of mesenchymal origin as they are also detected on primary skin fibroblasts (data not shown) and points out the lack of specific markers.

The multilineage potential of MSC from the synovium has already been described [6,7]. These studies only described qualitative results based on histological and immunohistological staining or semiquantitative RT-PCR. In our study, we performed quantitative RT-PCR to indicate quantitative differences in the expression level of the markers specific for the differentiated states. Variability in gene expression was observed between samples, independently of gender, age or the status of the patient (normal, OA or RA) (data not shown). In previous studies, the multilineage potential of synovium-derived MSC was reported to be independent of donor age, passaging or cryopreservation [7]. We now report that synovium-derived cells expressed significantly lower levels of osteogenic marker mRNA after *in-vitro*-induced osteogenesis, whereas these cells tended to secrete higher amounts of chondrogenic marker mRNA after chondrogenesis induction, although the differences with BM-derived MSC were not statistically significant. These data may reflect a different cellular phenotype or a different amount of MSC inside the two cell populations. Indeed, in the cells isolated from the synovium tissue we measured 1.8 ± 1.4 colony-forming units in 10^4 ^plated cells, which is in the same range obtained with cells from BM (estimated to be 1 in 10^4 ^or 10^5 ^mononuclear cells) [29]. Alternatively, they may reflect a commitment of stem cells under the influence of the environmental parameters. The presence of progenitors already committed to the chondrogenic lineage may be in higher amounts in the synovial membrane, where they contribute to the homeostasis of the cartilage tissue that is in close contact. Conversely, the higher capacity of differentiation toward osteoblasts observed with cells isolated from BM may suggest a higher numbers of cells committed to the osteogenic lineage inside the BM. Availability of markers specific for the MSC or different stages of differentiation would help to answer this question.

Another functional characteristic of MSC is their capacity to inhibit the proliferation of T cells in a MLR [15]. We show here for the first time that synovium-derived cells not only suppress the proliferative activity of T cells, but also exhibit functional IDO activity upon stimulation with IFN-γ to the same extent as BM-MSC. IDO activity has recently been suggested to contribute to the T-cell suppressive mechanism in human MSC. IDO has been identified as a T-cell inhibitory effector pathway in professional antigen-presenting cells upon induction by IFN-γ and other proinflammatory molecules such as TNF-α. This enzyme catalyzes the conversion from tryptophan to kynurenine; because tryptophan is an essential amino acid, its depletion will impair protein synthesis, leading to inhibition of cell proliferation. Depletion of tryptophan has also been shown to lead to stabilization of IL-6 and IL-8 mRNA, resulting in increased IL-6 and IL-8 responses that were proposed to be implicated in enhanced inflammatory responses to bacterial challenges after a viral infection [30]. A comparison of patients with RA, OA, psoriatic arthritis and gout recorded the highest levels of IL-1β, IL-6, IL-8 and IDO as well as the lowest levels of tryptophan in RA synovial fluids, indicating stimulated cellular immune responses in RA patients [31,32]. Indeed, the possible dual activity of IDO in synoviocytes as well as in BM-MSC still needs to be elucidated. In conditions where TNF-α and IFN-γ are only poorly present, the IDO activity may lead to an immunosuppressive environment inside the joint favoring the inhibition of immune cell proliferation. In the inflammatory context, where TNF-α and IFN-γ are prominent, induction of proinflammatory cytokines may reverse the cytokine balance, leading to a reversion of the immunosuppressive capacity of MSC as we previously showed in the collagen-induced arthritis model of arthritis [33].

The presence of MSC in the synovial membrane addresses the question of their origin and function within the joint. MSC in the synovium may be recruited from the blood that enters the synovial tissue, as they are present in normal conditions and even in higher numbers in the case of injury [8] due to their recruitment from the other tissues where they reside. MSC may also come from the bone marrow, which is connected with the intra-articular space by channels, enlarged in RA [10,34]. The role of MSC is possibly related to their potential to repair tissues of mesodermal origin present inside the joint in the case of traumatic or pathologic injuries [10]. Another postulated role for MSC is their possible involvement in the early phases of osteoarticular diseases and, in particular, in RA [10]. Although MSC possess immunosuppressive capacities, we have previously shown that they are unable to display a benefit in the collagen-induced arthritis model because they lose this property in the presence of TNF-α [33]. Moreover, the increase of MHC class II expression on MSC upon IFN-γ stimulation may further contribute to the aggravation of the immune response. We thus may postulate that TNF-α is the key molecule at the onset of RA pathogenesis that induces or contributes to modifying the characteristics of the MSC, which then act to favor the accumulation of immunocompetent cells into the joint.

An important feature revealed in the present study is that cells isolated from BM and the synovial membrane could be distinguished by distinct gene expression profiles. Both populations are thus characterized by the differential expression of various genes, in particular activin A that is upregulated in BM-MSC. Activins and inhibins are members of the transforming growth factor beta super-family that play roles in skeletal development and bone morphogenesis [23]. Activin A is a multifunctional cytokine that regulates cell growth and differentiation, whose effects are diverse depending on the cell type. In synoviocytes, activin A has been reported to promote proliferation, to be induced by IL-1β and to be upregulated in OA and RA patients [35-37]. It is still unclear, however, whether activin A accelerates or inhibits RA autoimmunity and inflammation. The secretion of activin A in BM-MSC is induced by BMP-2, at least *in vitro*, and therefore has been suggested to be downstream BMP-2 in the differentiation program that results in skeletal development [38]. In the bone marrow, where MSC are under the influence of transforming growth factor beta and BMP molecules, the upregulation of activin A may be involved early at the beginning of the cascade of events promoting chondrogenic/osteogenic differentiation [39]. However, activin A has been recently shown to play a role in the maintenance of the pluripotency of human embryonic stem cells [40]. The maintenance of pluripotency of embryonic stem cells could involve Wnt signaling and could occur through a crosstalk between the transforming growth factor beta/activin and Wnt pathways [41]. Indeed, activin A may play a dual role according to the environmental parameters: proliferation or maintenance of pluripotency. The higher amounts of activin A produced by BM-MSC together with similar numbers of colony-forming units further suggest their higher multipotent potential.

In summary, the similarity between adherent cells cultured from BM and from synovial tissue suggests a common origin. The few discrepancies between cells may reflect the impact of the tissue environment on the properties of MSC. Thus, due to pathological conditions, reduced differentiation properties and reversion of immunosuppression of MSC have been reported. It will be of therapeutic interest to determine whether MSC originating from various tissue sources share the same features. In this respect, the demonstration that high levels of activin A are produced by BM-MSC may potentially be of relevance in arthritis and repair since it may be associated with the pluripotency of the cells. MSC isolated from various tissues not involved in the specific pathology may be an alternative and more suitable source of cells with fully functional features for tissue engineering.

## Abbreviations

α-MEM = alpha-minimum essential medium; BM = bone marrow; BSA = bovine serum albumin; DMEM = Dulbecco's modified Eagle's medium; ELISA = enzyme-linked immunosorbent assay; FACS = fluorescent-activated cell sorting; FCS = fetal calf serum; IDO = indoleamine 2,3-dioxygenase; IFN = interferon; IL = interleukin; mAb = monoclonal antibody; MHC = major histocompatibility complex; MLR = mixed lymphocyte reaction; MSC = mesenchymal stem cells; OA = osteoarthritis; PBS = phosphate-buffered saline; PCR = polymerase chain reaction; RA = rheumatoid arthritis; RT = reverse transcriptase; TNF-α = tumor necrosis factor alpha.

## Competing interests

The author(s) declare that they have no competing interests.

## Authors' contributions

FD performed the majority of the experimental work and participated in the analysis of the data. CB participated in the cell culture work and the molecular analysis. TH procured the synovial fibroblasts from healthy donors. GU participated in the molecular analysis. NL performed the quantification of activin by ELISA. PL-P helped in the analysis of the data. FA helped in the analysis of the data. FC procured the samples of arthritic synovium. TR helped with the biostatistical analysis. JS participated in the analysis of the data. CJ participated in the design of the study and the analysis of the data. DN participated in the design of the study and the analysis of the data, and wrote the manuscript. All authors read and approved the final manuscript.

## Figures and Tables

**Table 1:** Genes whose mRNA expression is differentially expressed in bone marrow-derived or synovium-derived cells

Gene	Fold changes versus day 0
Upregulated in bone marrow-derived cells	
MIP-1β	40.5
IGF2	34.3
OX40 ligand	27.5
Neuregulin	26.8
Lymphotoxin-β	13.5
Acidic fibroblast growth factor	11.4
GAPDH	11.3
Lymphotoxin-α precursor	10.0
BIGH3	9.5
TRAIL	7.2
IFN-γ receptor	7.2
G-CSF	6.9
Glia-derived neurite-promoting factor (GDNPF)	6.4
IFN-α/β receptor β-subunit precursor	6.2
Hepatocyte growth factor	6.0
RPL13A	5.9
Platelet-activating factor receptor	5.6
Keratinocyte growth factor; FGF7	4.8
Endothelin receptor type A	4.5
IGF-binding protein 2 (IGFBP2)	4.1
Inhibin bA subunit precursor; activin bA subunit precursor	4.1
Apoptosis-related protein TFAR15	4.0
Related to receptor tyrosine kinase (RYK)	3.4
VEGFR1	3.3
IGF-binding protein 3 (IGFPB3)	3.0
Insulin-like growth factor I receptor (IGF1R)	2.9
Downregulated in bone marrow-derived cells	
MIP2-α, GRO-β	-26.2
IL-8 precursor	-25.3
Tumor necrosis factor-inducible protein TSG-6	-13.8
βNGF	-11.9
Granulocyte chemotactic protein (GCP2)	-6.2
FLT3 ligand	-5.1
Leukocyte IFN-inducible peptide	-4.9
BMP-4 + BMP-2B	-4.7
Activin receptor-like kinase 1 (Alk1); transforming growth factor beta superfamily receptor I (TSR1)	-4.2
Tumor necrosis factor alpha	-4.0
TRAIL receptor 2; death receptor 5	-3.8
BMP3B; GDF10	-3.6
Ciliary neurotropic factor receptor (CNTFR)	-3.6
Tumor necrosis factor receptor (TNFR)	-3.3
B-cell growth factor 1 precursos (BCGF1)	-3.2
IL-13 precursor	-3.1
Transforming growth factor beta	-2.8
IL-6	-2.6
ERBB2 receptor protein-tyrosine kinase	-2.5
IFN regulatory factor	-2.3

Upregulation or downregulation refer to the fold increase higher than ratio 2 in bone marrow samples (*n *= 4) compared with synovial samples (*n *= 4), with a Mann-Whitney significance of 0.05.
